# Primary inguinal subcutaneous endometriosis accompanied with an inguinal hernia: A case report

**DOI:** 10.1097/MD.0000000000025460

**Published:** 2021-04-09

**Authors:** Pei-Chen Chen, Chiu-Hsuan Cheng, Dah-Ching Ding

**Affiliations:** a Department of Obstetrics and Gynecology; b Department of Pathology, Hualien Tzu Chi Hospital, Buddhist Tzu Chi Foundation; c Institute of Medical Sciences, College of Medicine, Tzu Chi University, Hualien, Taiwan.

**Keywords:** cutaneous, endometrioma, inguinal hernia, primary, pubic

## Abstract

**Rationale:**

We report a case with inguinal subcutaneous endometriosis without typical cyclic dysmenorrhea and accompanied with a hernia sac treated with resection of the tumor and herniorrhaphy.

**Patient concerns:**

A 40-year-old woman had a painless enlarged inguinal nodule for 3 months.

**Diagnoses:**

Subcutaneous endometriosis accompanied with a hernia sac.

**Interventions:**

Ultrasonography showed a hypoechoic lesion (3.0 cm × 2.0 cm), and an inguinal subcutaneous tumor was first suspected. After surgical exploration, a cystic lesion was excised and the hernia hole was repaired by herniorrhaphy. The immunohistochemical analysis of the small endometriotic cyst-like lesion revealed calretinin (-) in epithelial cells and CD10 (+) in stromal cells, indicative of subcutaneous endometriosis accompanied with a hernia sac.

**Outcomes:**

The patient was followed up for 1 year and without recurrence.

**Lessons:**

Cutaneous endometriosis accompanied with a hernia sac can be presented without typical endometriosis-associated symptoms such as dysmenorrhea. Inguinal endometriosis might be the differential diagnosis of inguinal painless nodules.

## 1 Introduction

Extrapelvic endometriosis is a rare non-malformative gynecologic disease where the endometrial glands are present in extrapelvic locations such as the lungs, umbilicus, and peritoneum.^[1]^ Notably, cutaneous endometriosis is a rare condition (approximately 5% of endometriosis cases) that can be subdivided into 2 categories: primary (spontaneous) and secondary endometriosis.^[2]^ The pathogenesis for spontaneous endometriosis remains unclear, but the lack of preceding surgical history is its defining trait. Conversely, secondary cutaneous endometriosis is an iatrogenic result of a medical procedure. It is known to arise within surgical scars, usually in cesarean and gynecologic surgeries, such as hysterectomies, episiotomies, in laparoscopic trocar sites, and needle tracts created by amniocentesis.^[2–4]^

The first case of cutaneous endometriosis was reported in 1885 with very few subsequent literature reviews.^[5,6]^ The most common site of cutaneous endometriosis is at the umbilicus, while only 0.6% of cases appear in the inguinal region.^[7]^ The majority of these inguinal cases have a history of surgery that involves the uterine cavity. Due to its rarity, inguinal cutaneous endometriosis is often misdiagnosed as hernias or lipomas.^[7]^ Here, we report a case of inguinal subcutaneous endometriosis accompanied with a hernia sac in a patient without a surgical history that was treated by tumor excision.

## 2 Case report

The patient has obtained the written informed consent that the patient has given her consent for images and other clinical information to be reported in the journal.

A 40-year-old parous woman presented with a 3-month history of a painless persistent mass in the right inguinal region. The mass was slow-growing without cyclic symptoms during her menstrual period. Her menstrual cycle was regular without dysmenorrhea. The patient denied any medical and surgical history, including no previous history of instrumentation on the abdomen and inguinal regions.

**Figure 1:**
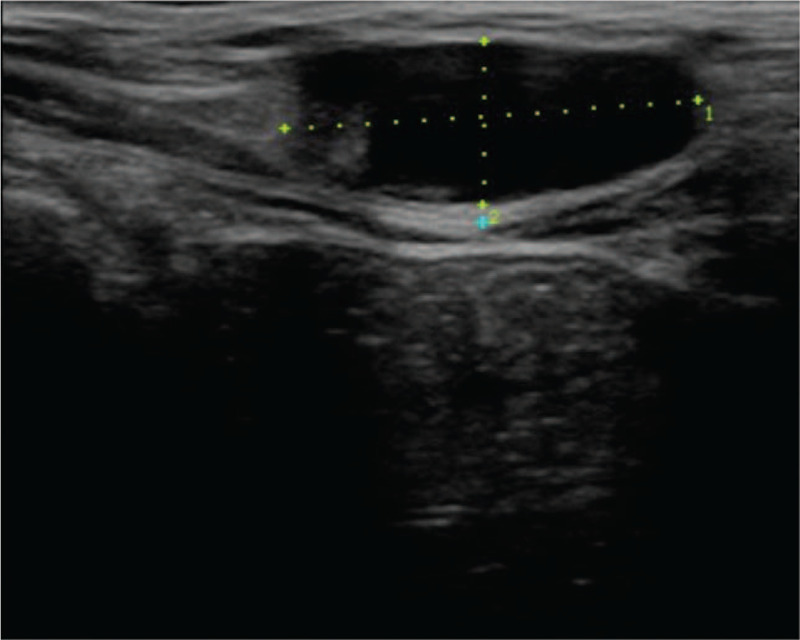
Ultrasound image of the pubic endometrioma showing a hypoechoic mass measuring 3.0 cm × 2.0 cm in the pubic region.

Upon physical examination, a painless, firm, and movable subcutaneous nodule was noted in the right inguinal region. Computed tomography (CT) was performed, disproving the possibility of an inguinal hernia. Abdominal ultrasound showed a right ovarian cyst and a homogeneous hypoechoic lesion (3.0 cm × 2.0 cm) in the right inguinal region (Fig. 1). We initially thought the mass was a subcutaneous tumor and thus tumor excision was advised.

**Figure 2:**
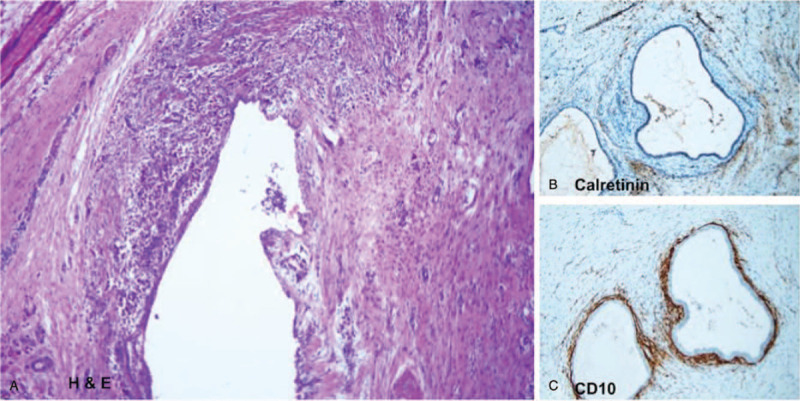
Histologic analysis of the pubic endometrioma. (A) Focal hemosiderin deposition in the hematoxylin & eosin (H & E)-stained specimen. (B) Calretinin (+) in the lining cells of the hernia sac. (C) The small endometriotic cyst-like lesion shows CD10 (+) in the stromal cells.

Under general anesthesia, tumor excision was done. During the procedure, a 3.0 cm × 2.0 cm subcutaneous tumor located in the suprapubic region containing clear fluid was found. After excisioning the tumor, a hernia hole was noted and repaired by herniorrhaphy. Pathology showed a hernia sac with focal hemosiderin deposition (Fig. 2A). Immunohistochemistry stain revealed the lining cells were positive for calretinin (Fig. 2B) and the stromal cells were negative for CD10. Conversely, the small endometriotic cyst-like lesion showed the expression of calretinin in the epithelial cells and CD10 in the stromal cells (Fig. 2C). Therefore, the patient was diagnosed with spontaneous subcutaneous endometriosis accompanied by an inguinal hernia.

The patient was followed-up as an outpatient for 1 year, and recurrent lesions and pain were not reported postoperatively.

## 3 Discussion

Inguinal endometriosis is a rare disorder occurring in only 0.6% of women.^[7]^ Due to its rarity, it is frequently misdiagnosed or overlooked. Patients with inguinal cutaneous endometriosis commonly have complaints of inguinal mass and pain that is usually aggravated during the menstrual cycle.^[7,8]^

The pathogenesis of inguinal cutaneous endometriosis remains unclear. Currently, there are 3 main theories of endometriosis pathogenesis: retrograde menstruation, which means menstruation contained endometrial cells retrograde move through the fallopian tube and into the peritoneal cavity; coelomic metaplasia, which designates normal peritoneal tissue transformation into ectopic endometrial tissue; and vascular or lymphatic spread, which denotes endometrial cells spreading through vascular or lymphatic tract.^[3]^ In our case, the endometrial cells might be accompanied with a hernia sac and move to the inguinal cutaneous region.

There are numerous possible causes for the development of an inguinal mass, and thus, differential methods of detection are crucial for correct diagnosis. Causes of inguinal mass include lymphadenopathy, hernia, abscess, neuroma, hematoma, hydroceles, lymphoma, lipoma, subcutaneous cysts, sarcoma, and cancer.^[9]^ In our case, we suspected subcutaneous lipoma first due to it being a movable and nontender mass.

Ultrasound and CT scan for the diagnosis of inguinal endometriosis are often not specific. Gaeta et al^[10]^ reported 2 patterns for inguinal endometriosis in magnetic resonance images (MRI). The first pattern consists of hyperintense cystic lesions on both T1- and T2-weighted images. The second pattern shows lesions having solid components – the T1-weighted images showing a high signal intensity, while the T2-weighted images showing hypointensity or moderate hyperintensity with some “shading signs” for cystic lesions.^[10]^ Although images can reveal some common patterns of endometriosis, accurate diagnosis relies on fine-needle aspiration cytology which is able to distinguish between benign and malignancy.^[11]^ If the cytology results indicate endometriosis, previous literature recommends tumor excision as the treatment method.^[12,13]^ In our case, we used ultrasound and CT scan to diagnose the mass. However, the specificity of both examinations is low.

Right side inguinal endometriosis was noted in over 90% of cases.^[7]^ Extraperitoneal round ligament is found to be the most affected portion by endometriosis and rarely associated with hernias. Previous reports showed 37% of patients with inguinal endometriosis accompanied with an inguinal hernia.^[14]^ Our case presented a right-side inguinal endometriosis with hernia representing the most common condition in the literature.

Recent studies have indicated that CD10 is a sensitive and diagnostically useful marker for the stroma of endometriosis.^[13,15]^ Therefore, in this study, we used CD10 as a marker for the endometriosis stroma. Mesothelial proliferation was noted in 6% of hernia sacs.^[15]^ The biological marker calretinin can identify mesothelial cells lining the hernia sac,^[16]^ and thus, we used calretinin as a marker to identify the hernia sac.

Surgical excision is the mainstay of the treatment for inguinal endometriosis.^[13]^ Complete excision of the lesion can prevent local recurrence. If the lesion accompanied an intrapelvic endometriosis, laparoscopic excision is indicated. Our patient received a complete surgical excision of a right inguinal endometriosis and hernia and repaired by herniorrhaphy.

Hormone therapy with oral contraceptives or gonadotropin-releasing hormone (GnRH) agonist can be another therapeutic modality. Oral contraceptives, pain killers, and GnRH agonist are used for pain relief.^[17]^ However, in our case, she did not experience cyclical pain. It may be due to small endometriosis lesions in the hernia sac region.

In conclusion, cutaneous endometriosis in the inguinal region is a rare disease usually seen in conjunction with menstrual cycle pain. Nevertheless, caution must be maintained when diagnosing an inguinal mass, as it is possible to not have any symptoms associated with menses, as seen in our patient. Ultrasound and CT are useful for evaluating the lesion, and MRI may also aid in identifying specific patterns. Surgical excision of the mass in inguinal endometriosis is the mainstay of treatment. Recurrence after surgical excision is rare.^[3]^ Our patient has no recurrence despite 1 year of follow-up.

## Author contributions

PCC: data analysis interpretation, manuscript preparation; CHC: pathological picture capture, manuscript preparation, DCD: study concepts, design and manuscript preparation, and revision.

**Conceptualization:** Dah-Ching Ding.

**Data curation:** Pei-Chen Chen, Chiu-Hsuan Cheng, Dah-Ching Ding.

**Formal analysis:** Pei-Chen Chen, Dah-Ching Ding.

**Investigation:** Dah-Ching Ding.

**Supervision:** Dah-Ching Ding.

**Writing – original draft:** Pei-Chen Chen, Dah-Ching Ding.

**Writing – review & editing:** Dah-Ching Ding.
